# A case report of spontaneous gastric perforation in a premature and low-birth weight neonate^☆^

**DOI:** 10.1016/j.ijscr.2024.109877

**Published:** 2024-06-06

**Authors:** Bradley Guidry, Christopher Prien, Kyle J. Glithero, Lynn Model, Daniel Hechtman

**Affiliations:** a Department of Surgery, Maimonides Medical Center, Brooklyn, NY, United States of America; b Division of Pediatric Surgery, Department of Surgery, Maimonides Medical Center, Brooklyn, NY, United States of America

**Keywords:** Spontaneous gastric perforation, Prematurity, Low-birth weight, Gastrorrhaphy, Stamm gastrostomy, Case report

## Abstract

**Introduction:**

Spontaneous gastric perforation of the neonate is a rare phenomenon with a high risk of mortality. Despite an uncertain etiology, an association with prematurity and low-birth weight has been demonstrated. Prompt surgical repair and intensive care remain imperative to survival.

**Presentation of case:**

A premature, low-birth weight male was born at 32 weeks and admitted to the NICU for respiratory distress syndrome. Forty-eight hours after birth he developed abdominal distention and an abdominal radiograph demonstrated pneumoperitoneum. Antibiotics were initiated and he was taken for emergent operative exploration. A 3 cm longitudinal perforation was identified in the greater curvature of the stomach. A two-layered repair was performed and a protective Stamm gastrostomy created. On postoperative day 10, an upper gastrointestinal contrast study demonstrated no evidence of leakage. After sustained clinical improvement, the initiation of oral feeding, and continued weight gain, the neonate was successfully discharged home.

**Discussion:**

The etiology of spontaneous gastric perforation remains a debate with several proposed mechanisms. In most cases, the neonate will present with abdominal distention and emesis. Although presentation and evidence of pneumoperitoneum on abdominal radiograph are suspicious for this pathology, definitive diagnosis is confirmed during operative exploration. Dedicated intensive care and prompt surgical repair are paramount to survival. Despite decreasing mortality rates, premature and low-birth weight neonates continue to have the lowest rates of survival.

**Conclusion:**

We present a rare case of a premature, low-birth weight neonate who developed spontaneous gastric perforation and was successfully rescued using a coordinated multidisciplinary approach enabling prompt diagnosis and surgical repair.

## Highlights

• Spontaneous gastric perforation is likely multifactorial in etiology due to numerous mechanisms.
• Gastrorrhaphy provides adequate repair of the perforation; protective gastrostomy should be strongly considered.
• Survival is reliant on dynamic intensive care and prompt surgical repair.

## 1 Introduction

Spontaneous gastric perforation of the neonate is a rare phenomenon affecting roughly 1:29,000 live births [1]. Overall, it accounts for approximately 10–16 % of all neonatal gastrointestinal perforations [1]. Although the etiology remains unclear its occurrence has been associated with prematurity and low-birth weight. Historically, mortality rates after spontaneous gastric perforation were as high as 100 % in neonates, but recently these rates have declined to as low as 16.7 % [2]. Despite this improvement, premature neonates with low-birth weight continue to have high rates of mortality with the smallest babies having the greatest rates of death. In this report, we present the rescue of a premature, low-birth weight neonate following spontaneous gastric perforation. This work is presented in accordance with the SCARE criteria [3].

## 2 Presentation of case

A male neonate weighing 1.55 kg was born prematurely at 32 weeks in a tertiary, academic institution. The baby was born vaginally after the mother went into preterm labor requiring artificial rupture of membranes. The pregnancy had otherwise been uncomplicated. At the onset of labor, betamethasone and ampicillin were administered. Following delivery, the baby appeared healthy with a spontaneous cry, an unremarkable physical examination, and one- and five-minute APGAR scores of 9 and 9 respectively. Laboratory studies at the time of birth exhibited a white blood cell count of 7.7 × 10 [9]/L, hemoglobin of 13.7 g/L, total bilirubin of 4.0 mg/dL, and electrolytes within normal ranges.

**Fig. 1:**
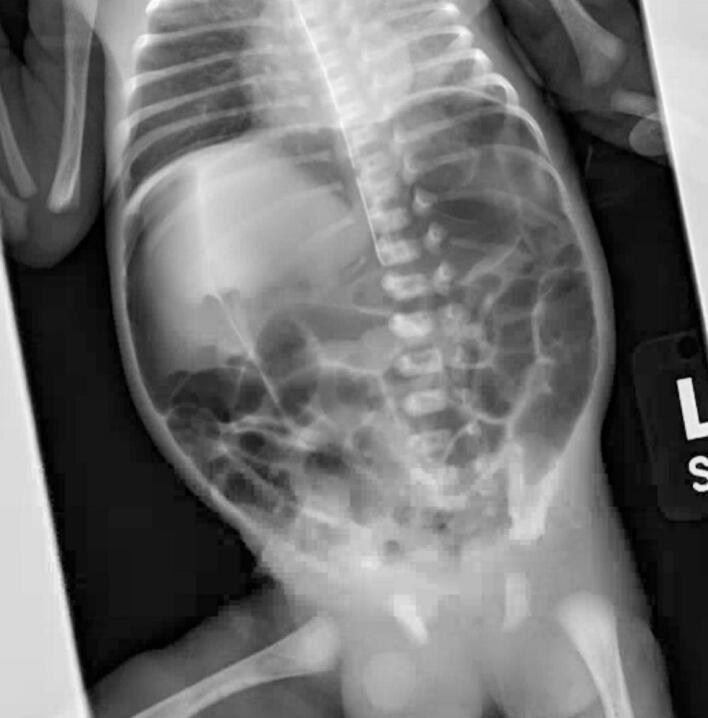
Abdominal radiograph demonstrating significant pneumoperitoneum.

He was subsequently admitted to the neonatal intensive care unit (NICU) for cardiorespiratory monitoring in the setting of respiratory distress syndrome and possible sepsis. Upon arrival to the NICU he remained hemodynamically stable but required supplemental oxygen via nasal continuous positive airway pressure (CPAP) and a Replogle tube was placed for gastric decompression. No transfusions were required. He was kept nil per os and was started on total parenteral nutrition. Forty-eight hours after birth, the neonate became noticeably distended and an abdominal radiograph was obtained. The radiograph demonstrated extensive pneumoperitoneum without evidence of intestinal pneumatosis (Fig. 1). Following prompt surgical evaluation, the neonate was started on intravenous antibiotics and taken to the operating room at the same institution as delivery for immediate surgical exploration.

Due to the initial concern for spontaneous intestinal perforation, typically found in the ileum, a right lower quadrant transverse incision was made. Intestinal evisceration failed to identify the site of perforation. The incision was carried across midline into the left lower quadrant to gain access to the remainder of the abdomen. Further exploration revealed a 3 cm longitudinal perforation along the proximal greater curvature of the stomach. The surrounding tissues appeared attenuated and ragged. The defect was repaired in two layers. A 10F Malecot Stamm gastrostomy was placed in the mid gastric body to provide gastric decompression during healing of the gastrorrhaphy. Postoperatively, the neonate returned to the NICU intubated and on intravenous antibiotics.

An upper gastrointestinal contrast study on postoperative day 10 to evaluate the integrity of the repair and demonstrated no evidence of leakage. The neonate was then weaned from the ventilator and successfully extubated on postoperative day 18. He was started on enteral feeds through the gastrostomy tube and subsequently transitioned to oral feeds. Prior to discharge the gastrostomy tube was exchanged for a low-profile gastrostomy tube and his weight had increased to 2.07 kg.

## 3 Discussion

Initially described by Siebold in 1825, the etiology of spontaneous gastric perforations in neonates has not been completely elucidated and is still under debate. Several mechanisms have been proposed as possible causes including congenital, ischemic, mechanical, drug-induced, and functional abnormalities, however no single cause has been identified across reports [4]. Despite this uncertainty, prior studies have demonstrated an association with prematurity and low-birth weight, with mortality rates more than 2-fold higher than those of normal-weight and full-term neonates [2,5]. Premature neonates have higher rates of lung immaturity and respiratory distress syndrome thus placing them at increased risk of hypoxia, need for supplemental oxygenation, and positive pressure ventilation, as well as poor feeding reflexes necessitating the placement of a nasogastric feeding tube. The neonate in this report exhibited a similar scenario.

One of the more intriguing mechanisms attributed to spontaneous gastric perforation is a congenital lack or underdevelopment of the gastric musculature. In a series by Yang et al. a total of 66 neonates with spontaneous gastric perforation were identified with 60 % found to have a lack of gastric musculature at the site of perforation [2]. The lack of musculature predisposed the neonatal stomach to perforation as it lacked the structural support necessary to accommodate distention or increased intra-gastric pressures. Interestingly the authors acknowledged the lack of musculature could have also been caused by fiber separation as the stomach distended or retraction of the musculature following perforation. Alternatively, Houck et al. described a functional abnormality due to a discoordinated peristaltic effort between the esophagus and stomach [6]. In that scenario, the stomach ultimately herniated into the mediastinum leading to ischemia and shearing along the greater curvature resulting in a linear perforation. In our neonate the tissue was thin and ragged, but with musculature surrounding the perforation. Microscopic evaluation of tissue from the site of perforation demonstrated an absence of muscle fibers.

Most cases of spontaneous gastric perforation in neonates occur within the first week of life. Abdominal distention is generally the initial presenting symptom and is frequently accompanied by emesis. The initial differential diagnosis will be broad, including necrotizing enterocolitis, septicemia, intestinal obstruction, or visceral perforation. An abdominal radiograph may demonstrate evidence of pneumoperitoneum. After prompt evaluation, resuscitation, and initiation of antibiotics, the neonate should be taken emergently for operative exploration. In most scenarios, gastrorrhaphy performed in a two-layered fashion will be a sufficient repair. When multiple perforations are encountered near each other, proceeding with a longitudinal sleeve gastrectomy has been shown to be safe and effective [7]. Alternative strategies, including wedge gastrectomy or gastrojejunostomy creation, should be avoided as they significantly alter the anatomy, and the creation of an anastomosis in a contaminated field during an emergent operation would be a significant risk for anastomotic leak. The use of gastrostomy tube should be individualized. Its benefit lies in the ability to protect the repair by ensuring gastric decompression and limited strain on the repair as it heals. Once healed, the gastrostomy tube can also be used to provide adjunctive enteral feeding access. Nasogastric decompression with a Replogle tube can be considered as an alternative to gastrostomy. While the stomach will remain decompressed during healing, the positioning of the tube can cause friction or place direct pressure on the site causing strain on the repair.

In a series by Rosser et al., a total of 13 neonates underwent repairs of spontaneous gastric perforations with gastrostomy creation with on a 7.5 % rate of mortality [8]. In contrast, Lin et al. reported their experience with primary gastrorrhaphy without gastrostomy in 15 neonates with a mortality rate of 47 % [5]. Although many factors influence outcomes, the contrast in mortality rates between these two studies suggests gastrostomy tubes may be beneficial. In premature, low-birth-weight neonates such as the one presented in this report, we believe the gastrostomy provided a survival benefit and should be strongly considered in this population as the risk of overwhelming sepsis and mortality is particularly high.

Despite adequate surgical repair, mortality remains high. Premature, low-birth weight neonates present a particularly difficult challenge as they are typically battling additional congenital and physiologic insults in addition to sepsis following perforation. Multiple reports have identified prompt surgical intervention, advancements in antimicrobials, and improvements in intensive care as paramount to minimizing mortality [2,9,10]. As a result, mortality rates have declined by over 80 % to as low as 16.7 % since the 1980s [2].

## 4 Conclusion

Spontaneous neonatal gastric perforation remains a condition with significant mortality. This case presents the rare rescue of a premature, low-birth weight neonate secondary to prompt diagnosis and intervention in which a Stamm gastrostomy was used to protect the repair. Continued improvements in outcomes will likely be tied to an improved understanding of etiology and treatments tailored to address the underlying causes in addition to prompt surgical repair.

## Parental consent

Written informed consent was obtained from the patient's parents for publication and accompanying images. A copy of the written consent is available for review by the Editor-in-Chief of this journal on request.

## Ethical approval

The institutional Ethics Committee waived the need for ethics approval for the collection, analysis, and publication of this anonymized, non-interventional study.

## Funding

This research did not receive any specific grant from funding agencies in the public, commercial, or not-for-profit sectors.

## Author contribution

Bradley Guidry: Conceptualization, Methodology, Administration, Writing – original draft, Writing – review & editing.

Christopher Prien: Conceptualization, Methodology, Administration, Writing – original draft, Writing – review and editing.

Kyle J. Glithero: Conceptualization, Methodology, Supervision, Writing – original draft, Writing – review & editing.

Lynn Model: Conceptualization, Methodology, Supervision, Writing – original draft, Writing – review & editing.

Daniel Hechtman: Conceptualization, Methodology, Supervision, Writing – original draft, Writing – review & editing.

## Guarantor

Dr. Daniel Hechtman.

## Research registration number

None.

## Conflict of interest statement

The authors declare that they have no known competing financial interests or personal relationships that could have appeared to influence the work reported in this paper.
